# Emergence of Norovirus GII.17 Variants among Children with Acute Gastroenteritis in South Korea

**DOI:** 10.1371/journal.pone.0154284

**Published:** 2016-05-05

**Authors:** Hien Dang Thanh, Van Thai Than, Tinh Huu Nguyen, Inseok Lim, Wonyong Kim

**Affiliations:** 1 Department of Microbiology, Chung-Ang University College of Medicine, Seoul 06974, South Korea; 2 Department of Pediatrics, Chung-Ang University College of Medicine, Seoul 06974, South Korea; University of Hong Kong, HONG KONG

## Abstract

Of 1,050 fecal specimens collected from January 2013 to August 2015 from children with acute gastroenteritis, 149 (14.2%) were found to be positive for norovirus. Norovirus GII was the most predominant genogroup (98.65%; 147 of 149). The genotypes detected in this study were GI (2; 1.3%), GII.Pe-GII.4 (109; 73.1%), GII.P17-GII.17 (16; 10.7%), GII.P12-GII.3 (8; 5.4%), GII.P12-GII.12 (8; 5.4%), GII.P4-GII.4 (5; 3.4%), and the recombinant GII.Pe-GII.17 (1; 0.7%). Of these, the novel GII.17 strain was the second most predominant, and the number of affected children appeared to continuously increase over time (2013 [2; 4.4%], 2014 [4; 9.3%], and 2015 [10; 16.4%]). Phylogenetic analysis of the full genome and ORF1, ORF2, and ORF3 nucleotide sequences showed that GII.17 was grouped in cluster III with other strains isolated from 2013 to 2015 and had a different evolutionary history from strains collected in 1978 to 2002 and 2005 to 2009 formed clusters I and II. However, the phylogenetic trees also showed that cluster III was divided into subclusters IIIa (CAU-55 and CAU-85) and IIIb (Kawasaki 2014) (CAU-193, CAU-265, CAU-267, CAU-283, and CAU-289). Comparative analysis of the VP1 capsid protein using 15 complete amino acid sequences from noroviruses isolated from 1978 to 2015 showed 99 amino acid changes. These results could be helpful for epidemiological studies to understand circulating norovirus genotypes in population.

## Introduction

Norovirus (NoV) is the predominant etiological viral agent of acute gastroenteritis across all age groups, but is most prevalent in young children and the elderly [1]. NoVs are recognized as the second most important cause of diarrhea in children following rotaviruses, and as an important cause of food-borne disease worldwide [2]. In most cases, NoVs cause diarrhea and vomiting, which generally last only a few days, but the symptoms can be serious for some people, especially young children and the elderly [1,3]. NoVs are responsible for an estimated 200,000 deaths among children aged less than 5 years each year in developing countries [4].

The genome of NoVs is a single-stranded positive-sense RNA approximately 7.7 kb in length that is covalently linked to VPg at the 5′ end and polyadenylated at the 3′ end. The genome is organized into three open reading frames (ORFs): ORF1 encodes for a polyprotein required for replication such as NTPase, protease, and RNA-dependent RNA polymerase (RdRp); ORF2 encodes the viral protein 1 (VP1); and ORF3 encodes the viral protein 2 (VP2) [5]. Based on genetic differences in the major capsid protein, NoVs are divided into at least seven genogroups, GI–GVI, of which GI and GII are the most frequently detected in human infections, and GIV is also implicated in human gastroenteritis [6]. Within each genogroup, strains are further subdivided into genotypes based on sequence information of the RdRp and capsid genes. GII strains are responsible for 75–100% of NoV cases worldwide, whereas GII.4 genotypes have been responsible for the majority of outbreaks, as well as community cases of acute gastroenteritis, since the mid-1990s [4,7,8].

Previous studies in South Korea have reported that 13–34% of the children admitted to hospitals with acute gastroenteritis or diarrhea were diagnosed with NoV infection. GII genotypes were recognized as predominant, and most of them belonged to GII.4, GII.3, GII.6, GII.8, and GII.13 [9,10,11]. Significant diversity of NoV genotypes has emerged with several novel recombinants, owing to the accumulation of point mutations or recombination resulting in the introduction of new antigenic variants [12].

Although several NoV strains have been in circulation over the last two decades, the novel GII.17, as the predominant outbreak strain in China, has recently been reported in several countries, including Japan, Hong Kong, Taiwan, the US, Australia, France, Italy, the Netherlands, New Zealand, and Russia [13,14,15,16,17,18]. In South Korea, an environmental samples study conducted from 2004 to 2006 indicated widespread circulation of GII.17, but the novel viruses themselves were not detected and there are no matching clinical reports [13]. The aims of this study were to: (i) genetically survey NoVs in South Korea during the years 2013 to 2015, (ii) genetically characterize the novel circulating NoV strain GII.17 isolated in children with acute gastroenteritis, and (iii) study the genetic evolution of GII.17. The data obtained in this study will be useful for researchers and will lead to a better understanding of NoVs, which could contribute essential information on the epidemiology and evolution of these viruses.

## Materials and Methods

### Ethics statement

The stool samples used in this study were collected and analyzed under protocol number #2010-10-02, approved by the Human Subjects Institutional Review Board (IRB) of Chung-Ang University College of Medicine, Seoul, Korea. For children enrolled in this study, written informed consent was got from parents or legal guardians. This consent included authorization to use the data for future research purposes.

### Stool sample preparation

From January 2013 through August 2015, 1050 stool samples were collected from children with acute gastroenteritis under 5 years of age. A case of acute gastroenteritis for the stool collection in this study was defined as increased stool frequency (i.e., at least three loose and watery stools within a 24-h period) with or without vomiting and fatigue occurring within the previous 48 h. Stool samples were collected from pediatric patients presenting with acute gastroenteritis at Chung-Ang University Hospital in Seoul. For sample processing, approximately 10% suspensions of stool samples were prepared by vortexing 0.1 g of stool sample with 1 mL of phosphate-buffered saline (PBS; pH 7.4). The stool sample suspensions were centrifuged at 12,000 × *g* for 15 min, and the supernatants were used as the fecal suspension.

### RNA extraction

Viral RNA was extracted from patient fecal suspensions using a QIAamp Viral RNA Mini Kit (Qiagen, Hilden, Germany) following the manufacturer’s instructions. Extracted viral RNA was stored at −70°C until use in reverse transcription-polymerase chain reaction (RT-PCR).

### NoV detection

The presence of NoV RNA was tested by RT-PCR using GI-specific primers (Calman-29 and Calman-32) and a semi-nested GII-specific primer set (Calman-1/Calman-2 for a first-round PCR and p289/p290 for a second-round PCR). RNA from NoV GII-positive specimens was analyzed by RT-PCR using a semi-nested GII-specific primer set (GIIF1M/GIIR1M for a first-round PCR and GIIF3M/GIIR1M for a second-round PCR) to amplify the capsid gene (Table 1). RT-PCR was performed using a One-Step RT-PCR Kit (Qiagen) under previously described amplification conditions [19,20]. PCR products were purified using a QIAquick PCR Purification Kit (Qiagen). The nucleotide sequencing was conducted by Macrogen (Seoul, Korea) using a BigDye Terminator Cycle Sequencing Kit and an automated DNA sequencer (Model 3730; Applied Biosystems, Foster City, CA, USA). Each amplicon was sequenced in both the forward and reverse directions. The sequences were assembled using BioEdit software (http://www.mbio.ncsu.edu/bioedit/bioedit.html). Preliminary genotypes were assigned by using the NoV genotyping tool (http://www.rivm.nl/mpf/norovirus/typingtool).

**Table 1:** Primers used for norovirus detection and full-length genome amplification.

Geno-types	Reaction type	Primer	Polarity^a^	Region	Location	Sequence (5'-3')	Reference
I	One-step RT-PCR	Calman-29	**+**	ORF1	4868–4891^b^	TATGGTGATGATGAAATAGTGTC	[19]
I	One-step RT-PCR	Calman-32	**-**	ORF1	5338–5356^b^	ATTTCGGGCAGAAGATTG	[19]
II	1st PCR	Calman-1	**+**	ORF1	4193–4213^c^	GCACACTGTGTTACACTTCC	[19]
II	1st PCR	Calman-2	**-**	ORF1	4997–5015^c^	ACATTGGCTCTTGTCTGG	[19]
II	Nested PCR	p290	**+**	ORF1	4568–4590^c^	GATTACTCCAAGTGGGACTCCAC	[19]
II	Nested PCR	p289	**-**	ORF1	4865–4886^c^	TGACAATGTAATCATCACCATA	[19]
II	1st PCR	GIIF1M	**+**	ORF2	5049–5067^c^	GGGAGGGCGATCGCAATCT	[20]
II	1st PCR	GIIR1M	**-**	ORF2	5367–5389^c^	CCRCCIGCATRICCRTTRTACAT	[20]
II	Nested PCR	GIIF3M	**+**	ORF2	5079–5102^c^	TTGTGAATGAAGATGGCGTCGART	[20]
II	Nested PCR	GIIR1M	**-**	ORF2	5367–5389^c^	CCRCCIGCATRICCRTTRTACAT	[20]
II	Full genome	NV-1F	**+**	ORF1	1–27^d^	GTGAATGAAGATGGCGTCTAACGACGC	[21]
II	Full genome	NV-1R	**-**	ORF1	2267–2287^d^	CACTATCTGRCACYTCTTGAT	[21]
II	Full genome	NV-2F	**+**	ORF1	2117–2139^d^	ACCTTCAAYTTTGACCGCAACAA	[21]
II	Full genome	JV13	**-**	ORF1	4594–4614^d^	TCATCATCACCATAGAAAGAG	[32]
II	Full genome	JV12	**+**	ORF2	4288–4308^d^	ATACCACTATGATGCAGATTA	[32]
II	Full genome	NV-3R	**-**	ORF2	6862–6881^d^	GCGCTTGGAGCATCTCTTTA	[21]
II	Full genome	COG2F	**+**	ORF3	5012–5037^d^	CARGARBCNATGTTYAGRTGGATGAG	[33]
II	Full genome	NV-4R	**-**	ORF3	7537–7556^d^	AAAAGATACAAATTAGCCAA	[21]

^a^ +, Forward primer; −, reverse primer.

^b^ Norwalk virus for GI (GenBank accession number M87661).

^c^ Lordsdale for GII (GenBank accession number X86557).

^d^ CUHK-NS-463 for GII.17 (GenBank accession number KP998539).

### Full-length genome amplification and sequencing

Seven out of sixteen GII.17-positive samples were selected for complete genome sequencing based on the quality and quantity of the available RNA: CAU-55 (collected in February, 2013); CAU-85 (collected in March, 2013); CAU-192 (collected in November, 2014); CAU-265 (collected in December, 2014); CAU-267 (collected in January, 2015); CAU-283 (collected in March, 2015); and CAU-289 (collected in April, 2015). To facilitate the sequencing of the full genome of the novel NoV strain, RT-PCR was performed using a One-Step RT-PCR Kit (Qiagen) with four pairs of primer sets (Table 1). RT-PCR amplification was performed at 42°C for 30 min, followed by 94°C for 1 min, and 35 cycles of PCR at 94°C for 30 s, 56°C for 30 s, 72°C for 2 min, and a final incubation at 72°C for 10 min [21]. Each PCR product was confirmed, purified, and sequenced as described above. The nucleotide sequence data were deposited in GenBank under accession numbers KU561250–KU561256.

### Phylogenetic analysis

The nucleotide sequences of NoV strains were aligned, and phylogenetic analysis was performed with other published reference strains obtained from GenBank database (http://www.ncbi.nlm.nih.gov/genbank) using the MEGA6 program suite [22]. The dendrograms were constructed using the neighbor-joining method with a bootstrap analysis of 1000 replicates in the MEGA 6.0 program.

### Statistical analysis

Continuous variables were analysed by the Student’s t-test. P value of < 0.05 were considered statistically significant. The tests were analyzed using Microsoft Excel 2010.

## Results

### NoV detection and molecular biology

**Fig 1:**
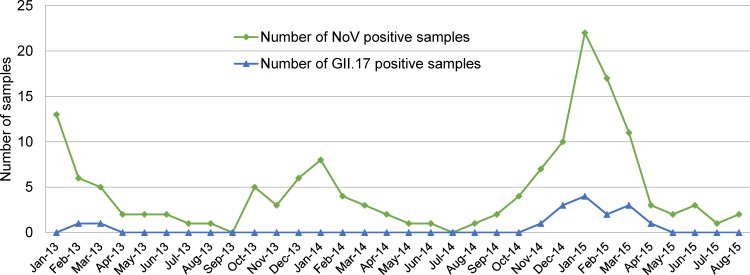
Monthly distribution of NoVs and genotype GII.17 in children with acute gastroenteritis from 2013 to 2015 in Seoul, Korea.

Overall, 1050 children hospitalized for acute gastroenteritis were enrolled in this study from January 2013 to August 2015. According to RT-PCR amplification and direct sequencing using the primers in Table 1, there were 149 NoV-positive cases, accounting for 14.2% of acute gastroenteritis cases. The GII genogroup accounted for most of the NoV-infected cases (98.7%, 147/149), followed by the GI strain (1.3%, 2/149). Sequence analysis of the RdRp gene in ORF1 and the capsid gene in ORF2 from 147 NoV GII-positive samples and a BLAST search revealed that these 147 samples could be divided into GII.Pe-GII.4 (109; 73.1%), GII.P17-GII.17 (16; 10.7%), GII.P12-GII.3 (8; 5.4%), GII.P12-GII.12 (8; 5.4%), GII.P4-GII.4 (5; 3.4%), and the recombinant GII.Pe-GII.17 (1; 0.7%) (Table 2). NoV outbreaks were detected year-round, but were mainly observed in the months of October through April. NoV GII.17 detections also occurred from November to March throughout the year, with peaks in January 2015 (Fig 1). Among the 149 NoV-positive cases, GII.17 was the second most predominant (n = 16) and accounted for 10.7% of these cases. The proportion of NoV GII.17 increased constantly from 4.4% in 2013 to 9.3% in 2014 and to 16.4% in 2015. In the NoV-positive patients hospitalized with acute gastroenteritis, there were no significant differences in clinical characteristics of patients infected with either the GII.4 or GII.17 genotype, such as daily diarrheal frequency with a mean of four liquid stools a day, diarrhea, abdominal pain, nausea, and fever (Table 3).

**Table 2:** Distribution of genotypes in the norovirus-positive samples in this study.

	2013	2014	2015	Total
No. Samples/No.Norovirus Positive (%)	346/45(13.0)	321/43(13.4)	383/61(15.9)	1050/149(14.2)
Norovirus genotype, n (% norovirus positive)				
GI	1 (2.2)	0	1 (1.6)	2 (1.3)
GII.Pe-GII.4	34 (75.6)	31 (72.0)	44 (72.2)	109 (73.1)
GII.P4-GII.4	0	2 (4.7)	3 (4.9)	5 (3.4)
GII.P17-GII.17	2 (4.4)	4 (9.3)	10 (16.4)	16 (10.7)
GII.P12-GII.3	4 (8.9)	2 (4.7)	2 (3.3)	8 (5.4)
GII.P12-GII.12	3 (6.7)	4 (9.3)	1 (1.6)	8 (5.4)
GII.Pe-GII.17	1 (2.2)	0	0	1 (0.7)

**Table 3:** Clinical information of children with NoV GII.17 and GII.4 genogroups infection.

Characteristic	GII.17 group (n = 16)	GII.4 group (n = 22)	P value
Age (months)	34 (18–58)	28 (14–58)	0.09
Male to female ratio	6:9	12:10	0.12
Frequency of vomiting (times/day)	2.1 (0–4)	2.6 (0–4)	0.12
Duration of vomiting (days)	2 (0–4)	1.6 (0–3)	0.15
Frequency of diarrhoea (times/day)	5.3(4–7)	4.8 (4–7)	0.07
Duration of diarrhoea (days)	3.7(2–5)	4.0 (2–6)	0.22
Fever of ≥ 37.5°C	11 (68.8)	16 (72.7)	0.22
Abdominal pain	75	66.7	0.29

## ORF analysis

From the NoV GII.17 specimens collected from January 2013 to August 2015, seven available specimens (CAU-55, CAU-85, CAU-192, CAU-265, CAU-267, CAU-283, and CAU-289) were selected for full-genome analyses. The NoV GII.17 genome is composed of three open reading frames: ORF1, ORF2, and ORF3.

**Fig 2:**
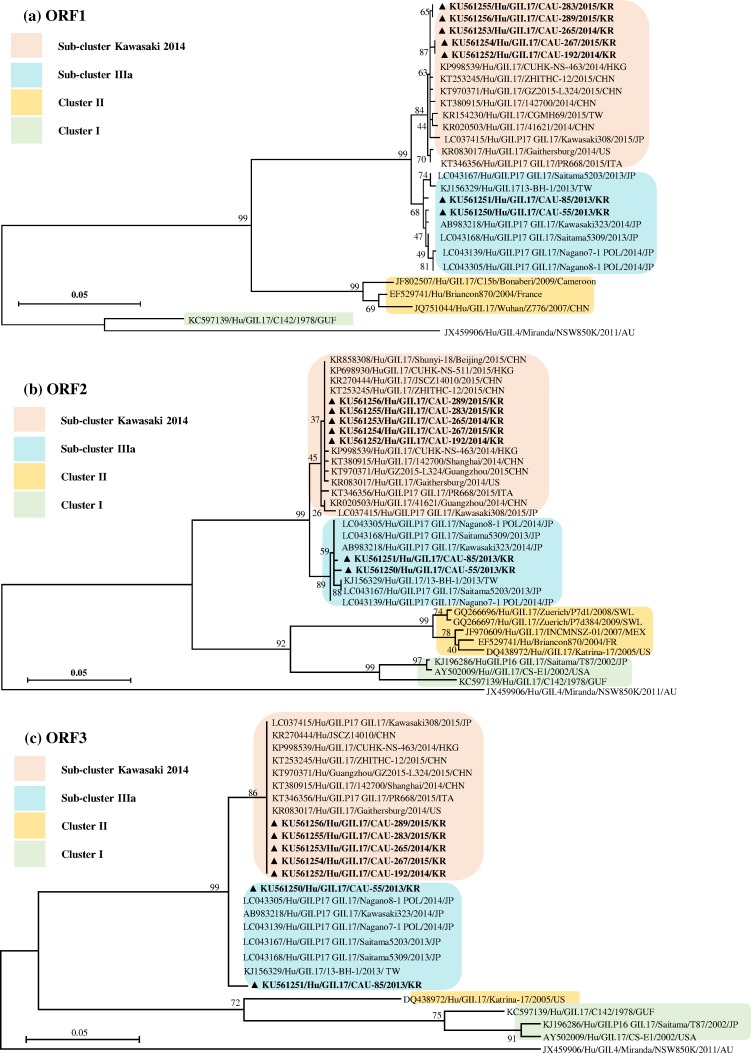
Phylogenetic tree based on the complete sequence of ORF1 (about 5.2 kb) (a), ORF2 (about 1.6 kb) (b), and ORF3 (about 0.8 kb) (c).

Phylogenetic analysis was performed with the target 5108-bp sequence of the ORF1 gene of the study strains and reference strains frequently isolated worldwide (S1 Table). As shown in the analysis of ORF1 (Fig 2A), cluster III contains the emerging GII.17 strain isolated in Japan, China, and the US from 2013 to 2015 as well as strains in this study, and the nucleotide sequences of the ORF1 genes show 98.1–99.8% identity with the reference strains. Although sharing a common ancestor in cluster III, the strains isolated in 2014 and 2015 are localized within Kawasaki 2014, whereas strains in 2013 formed a distinct branch inside subcluster Kawasaki 2014 and group with known GII.17 NoV strains from Japan and Taiwan isolated in the 2013/14 winter season.

Phylogenetic analysis of the ORF2 (VP1 protein) region was performed with the target 1623-bp sequence of the strains in this study and reference strains. As shown in Fig 2B, all strains isolated in South Korea were classified as members of cluster III, which was distinct from other strains isolated before 2011 (cluster II) and 2003 (cluster I). However, further diversification of the cluster strains led to two subclusters: subcluster IIIa comprised the CAU-55, CAU-85, and GII.17 strains from Japan and Taiwan in 2013–2014 (98.9–99.2% nucleotide sequence identity), whereas subcluster Kawasaki 2014 included the other five recent GII.17 strains identified in this study and strains isolated in Japan, China, the US, Italy, and Hong Kong from 2014–2015 (99–99.8% nucleotide sequence identity).

Phylogenetic analysis of ORF3 (VP2 protein) is described in Fig 2C. With respect to the ORF3 780-bp region, the CAU-55 and CAU-85 strains showed the highest similarity with the Kawasaki323 and 13-BH-1 strains, exhibiting 98.2–99.3% and 95.7–98.9% nucleotide and amino acid sequence identities, respectively. The other five recent GII.17 strains shared high sequence identity with ZHITHC-12, Kawasaki308, and Gaithersburg (98.8–99.7% and 97.7–100% nucleotide and amino acid sequence identities, respectively). Interestingly, high sequence identity was observed among strains isolated in 2014 and 2015 (99–99.7%), but GII.17 strains isolated in 2013 displayed only 96.7% and 96% nucleotide and amino acid sequence identities, respectively.

### Phylogenetic analysis of the NoV genome

**Fig 3:**
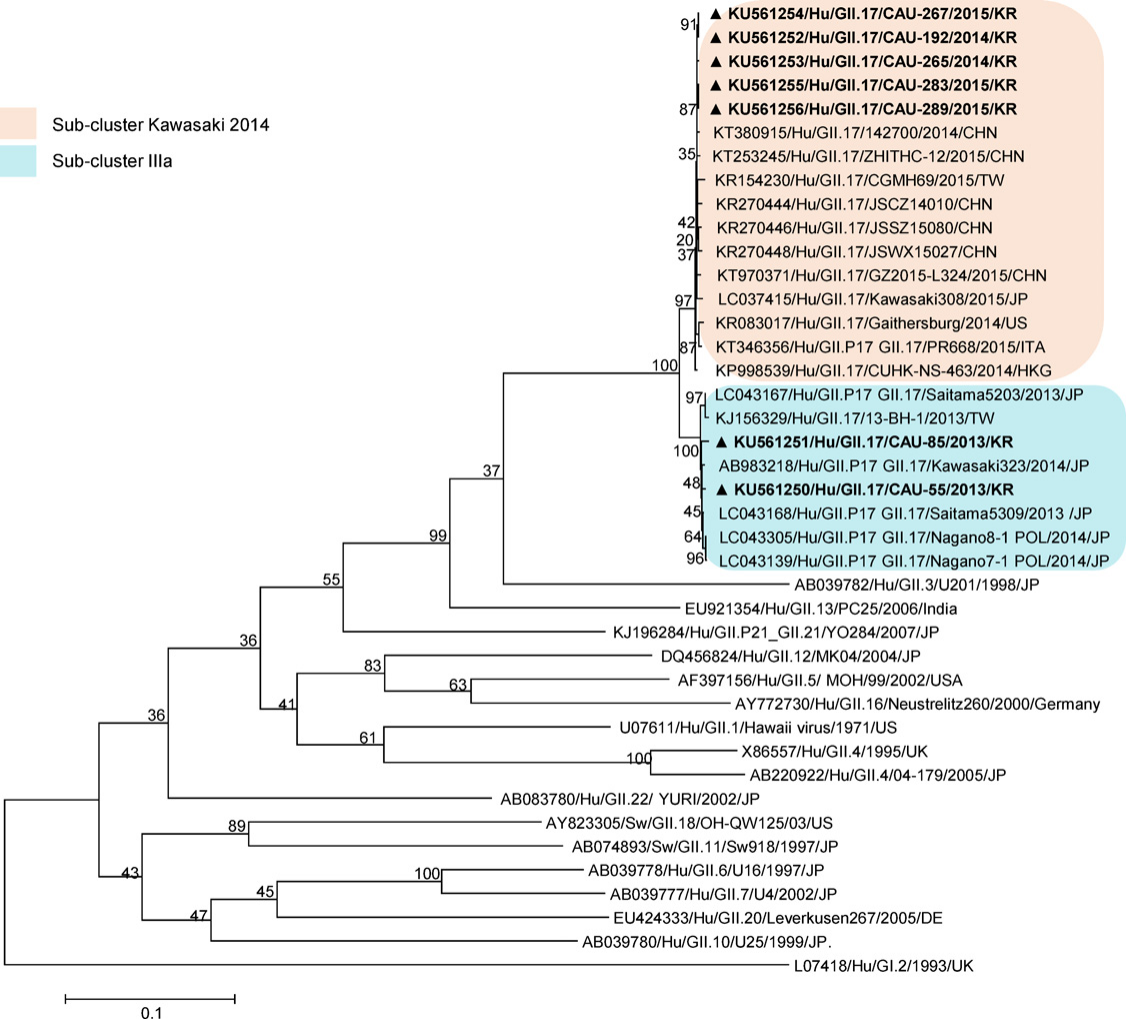
Phylogenetic analysis based on the full (or near-full-length) ORF1 and ORF2 gene. GI.1 strain was used as outgroup. Strains analyzed in the present study are indicated by a black triangle.

For phylogenetic analysis of the NoV genome, NoV GII.17 full-genome sequences obtained in this study from South Korea between 2013 and 2015 (n = 7), reported GII.17 reference sequences of past global epidemics, and representative sequences of the non-GII.17 genotypes reported to date in the GenBank database were used (S1 Table). As observed in the maximum-likelihood ORF1 and ORF2 tree (Fig 3), most of the non-GII.17 sequences were located far from the GII.17 cluster, and NoV GII.17 strains genetically formed the same cluster. Within the GII.17 cluster, the 2013/14 season strains were grouped separately from the GII.17 strains detected in 2014/15. The 2014/15-season strains were genetically related (99.8%) and clustered with the GII.P17-GII.17 strain detected in the US, Italy, and Asia in the 2014/15 seasons; these belonged to the novel NoV GII.17. It is interesting to note that the GII.17 strains are genetically most similar to GII. 3 reference strains.

### Amino acid variation in the viral structural protein VP1

**Fig 4:**
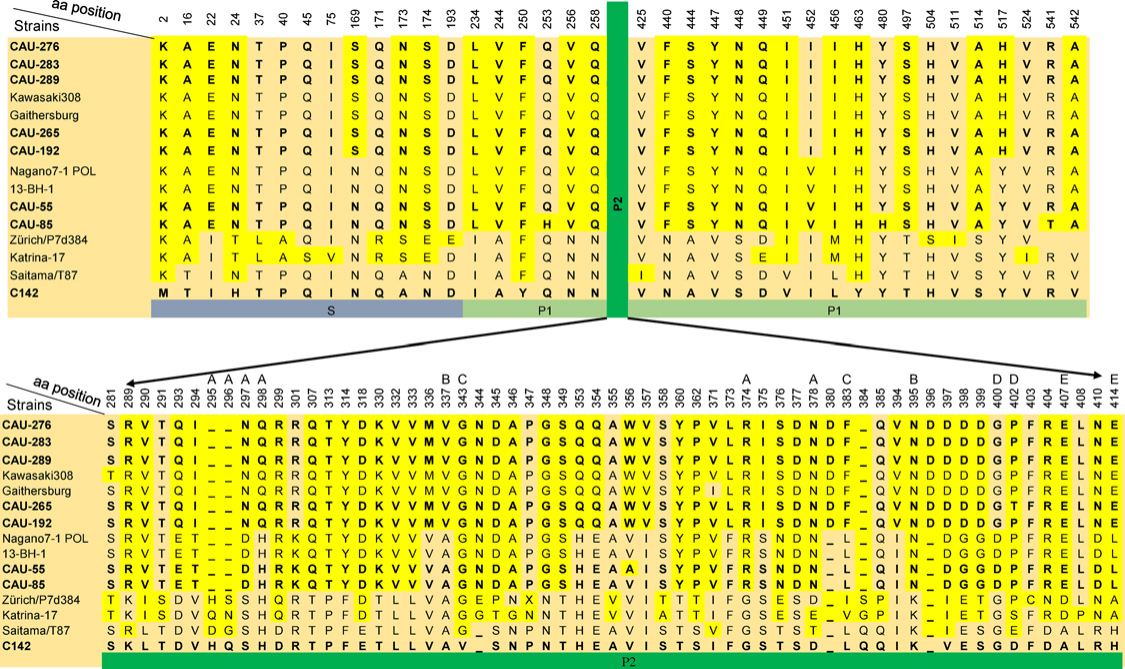
Amino acid substitutions in the VP1 sequence of norovirus GII.17 strains over time. The putative blockade epitopes A–E are indicated. Dashes indicate deletions/insertions of the amino acid residues. Amino acid numbering is based on the sequence of the C142 strain (JN699043).

Seven GII.17 capsid protein VP1 sequences in this study were aligned with representative viruses of the other GII.17 clusters isolated from 1978 to 2015, including one or two representative strains from each of three clusters previously released and deposited in GenBank. Alignment of the derived amino acid sequences revealed 99 variable residues across the VP1 domain of the study strains as compared to the strains in the other two clusters, representing up to 18% of the total VP1 amino acids; their locations in the structure are shown in Fig 4. Among the 99 variable amino acids, 13 (13.13%) were observed in the shell domain and 25 (25.25%) in the P1 region; most of the substitutions and insertions were located in the P2 region, which contains the antigenic epitopes and host receptor-binding domain. In the capsid-protruding hypervariable P2 region, 60 amino acids accumulated changes at several positions, including epitope A amino acids 297–298, 374, and 378; epitope B amino acids 337 and 395; epitope C amino acid 383; epitope D amino acids 400 and 402; epitope E amino acids 407 and 414; and 1 substitution at epitope C amino acid 343. Changes in these residues likely alter the ability of preexisting immunity to neutralize the virus, thereby facilitating the emergence of new epidemic strains.

Compared with the GII.17 strains identified before 2013/14, mutations were observed in the RGD/K-like motif (K289R), located at positions 287–289 of five recent strains. Three deletions (residues 295, 296, and 384) and one insertion (344) of all the strains in this study differed markedly from the oldest GII.17. Of note, the 2014/15 winter season strains contained 2 amino acid insertions (-380D and -396D; C142 numbering) in the P2 region as compared to the previous strains, resulting in different lengths of VP1. Among cluster III, five strains in the 2014/15 winter season presented 25 amino acid site substitutions (sites N169S, E293Q, T294I, D297N, H298Q, K301R, V336M, A337V, H353Q, E354Q, I357V, F373L, S375I, N376S, -380D, L383F, I394V, -396D, G398D, G399D, D400G, D410N, L414E, V452I, and Y517H) and accumulated changes at five positions (H253Q, A356W, P402T, H480Y, and T541R) as compared with viruses isolated in 2013 (CAU-55 and CAU-85).

## Discussion

Enteric viruses such as rotaviruses, NoVs, astroviruses, and adenoviruses are the most significant etiological agents of childhood viral gastroenteritis in both developing and developed countries [23]. NoV is considered to be the second most common cause of acute gastroenteritis requiring hospitalization among children below five years of age [2]. Laboratory-confirmed NoV infections involving 149 strains (14.2%) were isolated from children with acute gastroenteritis in this study. Similar findings have been reported with a figure of 13% in patients with acute gastroenteritis symptoms from five participating hospitals in three regions (Seoul, Gyeonggi Province, and Gangwon Province) from 2007 to 2010 [10]. However, this was a lower percentage of NoV-positive samples than those reported in previous studies [9,11]. This discrepancy might be due to differences in the duration of the studies, the study design, and the target population, for example.

Six different NoV genotypes were identified by BLAST using 300-bp sequences of the polymerase and capsid genes. The predominant genotype in this study was NoV GII.4, which accounted for 114 of 149 (76.5%) of the detected NoV strains. Several other previous studies in Korea have also shown that most of the NoV GII genotypes belonged to GII.4, followed by GII.3, GII.6, and GII.8 [9,24,25]. However, the relatively high detection rate (10.75%) of the previously rare GII.17 genotype reported in South Korea indicated changing patterns of the circulating NoV strains. Although it is still too early to predict whether NoV GII.17 will replace NoV GII.4, GII.17 is the predominant genotype in Asian countries such as Hong Kong and Taiwan, and in Japan the number of cases caused by this novel virus sharply increased from 2013 to 2015 [13].

Phylogenetic analysis of the full-length sequences established a separate cluster for the NoV GII.17 strains in a sister relationship with other known NoV GII.17 strains identified earlier. NoV GII.17 strains in Korea had evolutionary relationships with those obtained from 2013–2015 in the US, Italy, China, Japan, and Hong Kong. However, it is surprising to note that the isolated Korean NoVs diverged into two distinct subclusters within a relatively short period of time. The rapid evolution of the novel GII.17 strain may lead to it evading the host immune response, driving changes in histo-blood group antigen affinities, and altering population susceptibility patterns [26,27,28]. Interestingly, the GII.3 strains detected in the 2000s have an evolutionary relationship with the recent GII.17 cluster III viruses.

Analysis of VP1 protein sequences revealed 99 amino acid changes. VP1 is further divided into the shell (S) comprised of amino acids 1–220 and a protruding (P) domain comprised of amino acids 221–541. The S domain contains elements essential for formation of the contiguous shell of the virus [29]. The P domain is divided into two subdomains, P1 and P2 [5]. P1, a conserved region among NoV strains across genogroups, consists of amino acid residues 221 to 276 and 415 to 541. P2, which consists of amino acids 277 to 414 as an insertion into P1, is hypervariable in sequence. Meanwhile, the P2 region is thought to play an important role in receptor binding and immune reactivity, and is likely to be primarily responsible for ABO histo-blood group antigen interactions associated with susceptibility to NoV infections [14,30]. Mutations in this region may have a significant effect on binding between this protein and carbohydrate antigens, and on potential downstream cooperative binding interactions [26,29,31]. Comparison of amino acid sequences between Korean NoV strains of 2013 and 2014/15 revealed the accumulation of mutations at several sites in VP1, and particularly in the main blockade epitopes located in the P2 region.

## Conclusions

The present findings demonstrate that the emerging new variants of NoV GII.17 were the second most predominant in all NoV-positive children with acute gastroenteritis. Amino acid analysis revealed several mutations in the P2 region of the recent strains. Thus, it is necessary to continue epidemiological surveillance of the emergence of GII.17 and monitor trends in its geographical spread and evolution.

## Supporting Information

S1 Table The norovirus in this study and reference strains used to construct phylogenetic tree. (DOC)
