# Supplementary material for: Emergence of Norovirus GII.17 Variants among Children with Acute Gastroenteritis in South Korea
Source: PLoS One. 2016 May 5;11(5):e0154284. doi: 10.1371/journal.pone.0154284 (PMC4858242; doi:10.1371/journal.pone.0154284)
Supplement: S1 Table — (DOC) [file pone.0154284.s001.doc]

**Emergence of Norovirus GII.17 Variants among Children with Acute Gastroenteritis in South Korea**

Hien Dang Thanh^1^, Van Thai Than^1^, Tinh Huu Nguyen^1^, Inseok Lim^2^, Wonyong Kim^1^,*

^1^ Department of Microbiology, Chung-Ang University College of Medicine, Seoul 06974, South Korea

^2^ Department of Pediatrics, Chung-Ang University College of Medicine, Seoul 06974, South Korea

***Corresponding author**

E-mail: [kimwy@cau.ac.kr](mailto:kimwy@cau.ac.kr) (WK)

Table S1. The norovirus in this study and reference strains used to construct phylogenetic tree

| Strain name | Genotype | Genbank accession number |
| --- | --- | --- |
| CAU-55 | GII.17 | KU561250 |
| CAU-85 | GII.17 | KU561251 |
| CAU-192 | GII.17 | KU561252 |
| CAU-265 | GII.17 | KU561253 |
| CAU-267 | GII.17 | KU561254 |
| CAU-283 | GII.17 | KU561255 |
| CAU-289 | GII.17 | KU561256 |
| C142 | GII.17 | KC597139 |
| Saitama/T87 | GII.17 | KJ196286 |
| CS-E1 | GII.17 | AY502009 |
| Briancon870 | GII.17 | EF529741 |
| Katrina-17 | GII.17 | DQ438972 |
| Wuhan/Z776 | GII.17 | JQ751044 |
| INCMNSZ-01 | GII.17 | JF970609 |
| Zuerich/P7d1 | GII.17 | GQ266696 |
| Zürich/P7d384 | GII.17 | GQ266697 |
| C15b/Bonaberi | GII.17 | JF802507 |
| Nagano7-1 POL | GII.17 | LC043139 |
| Saitama5203 | GII.17 | LC043167 |
| 13-BH-1 | GII.17 | KJ156329 |
| Kawasaki323 | GII.17 | AB983218 |
| Saitama5309 | GII.17 | LC043168 |
| Nagano8-1 POL | GII.17 | LC043305 |
| Kawasaki308 | GII.17 | LC037415 |
| 41621 | GII.17 | KR020503 |
| PR668 | GII.17 | KT346356 |
| Gaithersburg | GII.17 | KR083017 |
| GZ2015-L324 | GII.17 | KT970371 |
| 142700 | GII.17 | KT380915 |
| CUHK-NS-463 | GII.17 | KP998539 |
| ZHITHC-12 | GII.17 | KT253245 |
| JSCZ14010 | GII.17 | KR270444 |
| CUHK-NS-511 | GII.17 | KP698930 |
| Shunyi-18 | GII.17 | KR858308 |
| CGMH69 | GII.17 | KR154230 |
| Miranda/NSW850K | GII.4 | JX459906 |
| U201 | GII.3 | AB039782 |
| PC25 | GII.13 | EU921354 |
| MK04 | GII.12 | DQ456824 |
| Neustrelitz260 | GII.16 | AY772730 |
| MOH/99 | GII.5 | AF397156 |
| YO284 | GII.21 | KJ196284 |
| Hawaii virus | GII.1 | U07611 |
| Hu/GII.4/1995/UK | GII.4 | X86557 |
| 04-179 | GII.4 | AB220922 |
| YURI | GII.22 | AB083780 |
| OH-QW125 | GII.18 | AY823305 |
| Sw918 | GII.11 | AB074893 |
| U4 | GII.7 | AB039777 |
| U16 | GII.6 | AB039778 |
| Leverkusen267 | GII.20 | EU424333 |
| U25 | GII.10 | AB039780 |
| Hu/GI.2/1993/UK | GI.2 | L07418 |
